# The relationship between quality of life and coping strategies of children with EB and their parents

**DOI:** 10.1186/s13023-021-01702-x

**Published:** 2021-01-30

**Authors:** Petra J. Mauritz, Marieke Bolling, José C. Duipmans, Mariët Hagedoorn

**Affiliations:** 1 Department of Dermatology, University Medical Center Groningen, University of Groningen, Hanzeplein 1, 9700 RB Groningen, The Netherlands; 2 Department of Health Psychology, HPC FA12, University Medical Center Groningen, University of Groningen, POB 196, 9700 AD Groningen, The Netherlands

**Keywords:** Quality of life, Coping strategy, Parent–child dyad, Epidermolysis bullosa

## Abstract

**Background:**

Epidermolysis bullosa (EB) is a group of rare genetic skin disorders that primarily manifest as blisters and erosions following mild mechanical trauma. Despite the crucial role of the parents of children with EB in managing the disease, studies focusing on the parent–child relationship remain a gap in the literature. To address this gap, the current quantitative study, involving 55 children with all types of EB and 48 parents, assessed the relationship between their quality of life and coping strategies. Quality of life was measured with the Pediatric Quality of Life Inventory and TNO-AZL Questionnaire for Adult’s Health- related Quality of Life, and coping strategies were assessed with the Coping with a Disease Questionnaire. The majority of the analyses were descriptive and the results were interpreted qualitatively because of the small sample size.

**Results:**

Overall, the quality of life of children with EB and that of their parents was somewhat lower compared with the quality of life of healthy children and adults. Children with EB who more frequently used emotional reactions and cognitive-palliative strategies to cope with the disease demonstrated lower levels of emotional and social functioning, while children who showed more acceptance and distancing showed higher levels of functioning on all domains. Parents who frequently demonstrated emotional reactions reported lower levels of social functioning and experienced more depressive emotions and anger. Parents who used more avoidance showed higher levels of positive emotions. Within parent–child dyads, acceptance, cognitive-palliative strategies and distancing were positively related. Children’s emotional and social functioning were negatively associated with their parents’ depressive emotions. Parents’ acceptance was linked to higher physical functioning in children, whereas children’s avoidance was linked to a lower level of anger in parents.

**Conclusion:**

Children who are able to accept the disease or distance themselves from it appear to be better off in contrast to those who tend to engage in the cognitive-palliative strategies and expressing emotional reactions. Parents seem to be better off when they are able to use avoidance in contrast to those who tend to show emotional reactions. Further research is needed to substantiate these findings.

## Background

*Epidermolysis bullosa* (EB) is a group of rare genetic skin blistering disorders. The condition primarily manifests as blisters and erosions following mild mechanical trauma. EB is categorized into four main types according to cleavage levels within the skin: EB simplex (EBS) with intra-epidermal blister formation; junctional EB (JEB), entailing skin cleavage at the level of the lamina lucida; dystrophic EB, in which there is blistering at the level of the sublamina densa; and Kindler syndrome, which may exhibit varying levels of blister formation [1]. The severity of EB is highly variable, ranging from relatively minor skin fragility to severe forms with lethal outcomes within the first weeks or months of life [2]. EBS may be dominant or recessive, associated with varying degrees of severity, ranging from mild blistering that only affects the hands and feet to severe generalized skin blistering with inflammation and the formation of palmoplantar keratoderma. Wound healing without scarring and significant clinical improvement during puberty are typical characteristics of EBS. JEB is always recessive and can be life threatening, manifesting in more severe symptoms, often characterized by widespread blistering and wounds; wound healing problems with hypergranulation; and hair, nail, mucosal, and laryngeal involvement. DEB varies from a mild dominant form, entailing lesions confined to the hands, knees, and elbows to a severe recessive form characterized by generalized blistering, scar formation with pseudosyndactyly, esophageal strictures, and the development of aggressive squamous cell carcinoma with reduced life expectancy. Kindler syndrome is characterized by blistering in childhood that is followed by poikiloderma, mucosal involvement, and light sensitivity in later life. The condition generally develops at birth or soon after birth, and the prognosis depends on the type of EB present. Some forms may include extracutaneous involvement that is either secondary, caused by severe mucocutaneous blistering, or primary, resulting from a genetic defect [3–7]. Pain and itching are notable characteristics and invalidating effects of the disease. There is currently no cure for EB, and treatment focuses on symptom relief and wound prevention [8].

Several studies indicate that EB affects the quality of life of patients with varying degrees of impairment of their physical, social, and psychological functioning [8–12]. Studies that have specifically focused on children with EB have revealed physical and psychosocial impairments, such as pain or itching, a sense of the self as “wrong,” others’ lack of understanding, or difficulties with social participation [13–15]. EB can also inflict a heavy burden on family members, caused by immense practical and psychological demands that include resource-intensive care and coping with complex feelings [10, 12, 16–18].

Coping strategies appear to be an important psychosocial moderator between stress and quality of life in chronic illness [19]. Coping can be defined as purposeful, volitional efforts that are directed at the regulation of aspects of the self and the environment under stress [20, 21]. There is considerable evidence that chronically ill children’s coping strategies, including problem solving, cognitive reappraisal (i.e., the changing of underlying appraisals that contribute to negative emotions) [22], positive thinking, and acceptance enable them to adjust better to their illnesses [23]. In contrast, denial, social withdrawal, and wishful thinking were found to be strongly linked to anxiety, sadness, and somatic complaints [24, 25]. Among the parents of (chronically) ill children, acceptance and seeking social support from friends and family [26], maintaining an optimistic attitude toward the caregiving situation [27], and the use of certain problem-focused coping behaviors have been linked to lower parental stress [28], while self-blame, wishful thinking, and avoidance were found to be associated with maternal maladjustment [29–31]. However, despite the findings of previous studies that parents and children influence each other’s behavior and well-being [32–34], few studies have investigated the relationships between the coping strategies of children and parents and their own as well as each other’s quality of life.

Although parents play a crucial role in disease management, given the daily necessity of care, to the best of our knowledge, no studies have focused on the parent–child relationship. The current study was aimed at addressing this gap by focusing on parent–child dyads. We examined the quality of life and coping strategies of both children with EB and their parents. Specifically, we investigated which coping strategies used by children and their parents were associated with their own quality of life. Moreover, we examined associations between the quality of life of children and psychosocial aspects of the quality of life (i.e., social and emotional functioning) of their parents as well as associations between children’s and their parents’ coping strategies. We further investigated which of the coping strategies deployed by children were related to the psychosocial aspects of their parents’ quality of life, and which of the parents’ coping strategies were associated with their children’s quality of life.

## Materials and methods

### Participants

We applied the following inclusion criteria for participants: (1) patients with EB simplex (EBS), junctional EB (JEB), dominant or recessive dystrophic EB (RDEB or DDEB), or Kindler syndrome (KS) aged < 25 years and registered as patients at the University Medical Center of Groningen (UMCG) in the Netherlands; and (2) parents of patients with EBS, JEB, RDEB, DDEB, or KS aged < 25 years and registered at the UMCG, who most frequently attended to their children’s wound care. We sent out invitation letters to parents of all 124 children aged < 25 years registered at the UMCG. The patients (hereafter called “children”) included young adults aged 18–25 years because of the small population of patients with EB in the Netherlands and the involvement of most parents in the care of their children beyond adolescence.

### Procedure

After the participants had provided their informed consent, they were invited to complete an online questionnaire that was accessible through a secure web portal. If there was more than one child with EB in a family, only the oldest child was included in the study. A reminder mail was sent to participants who had not completed questionnaires after 1 month. Different sets of questionnaires were sent to children and to parents (see “Materials and methods”).

### Materials

Children aged 8 years or older independently completed questionnaires about their quality of life and coping strategies. If the child was between 2 and 8 years, parents completed questionnaires about the child’s quality of life, but not their coping strategies. We did not assess the quality of life and coping strategies of children younger than 2 years. All of the participating parents completed questionnaires about their own quality of life and coping strategies.

### Quality of life of children

We assessed the quality of life of children aged 2–25 years using the Pediatric Quality of Life Inventory (PedsQL). This instrument evaluates the health-related quality of life of children with chronic illnesses from the perspective of the child/young adult (8–25 years) or that of the parents of younger children (2–7 years). There are age-appropriate versions of the questionnaire for the following age groups: 2–4, 5–7, 8–12, 13–18, and 18–25 years. The respondents were instructed to report how big a problem a specific item had been for the child during the past month. The 23 items were scored using a 5-point scale, ranging from 0 (“not a problem”) to 4 (“a problem almost all of the time”). There are four subscales representing different dimensions of quality of life: (1) physical functioning (eight items, e.g., pain), (2) emotional functioning (five items, e.g., feeling angry), (3) social functioning (five items, e.g., problems with peers) and school functioning (five items, e.g., difficulties with concentration). Good reliability and validity has been reported for both the American [35] and the Dutch version of the PedsQL [36].

### Quality of life of parents

Parents’ quality of life was assessed with the TNO-AZL Questionnaire for Adult’s Health-related Quality of Life (TAAQoL). This questionnaire measures an individual’s health status problems experienced over the last month, which are weighted by the impact of these problems on his or her well-being. The instrument consists of 12 multi-item scales: gross and fine motor functioning, cognitive functioning, sleep, pain, social functioning, daily activities, sexuality, vitality, positive emotions, depressive emotions, and anger. The raw scores were converted to a 0–100 scale, with higher scores indicating better QoL. The validity and the reliability of the TAAQoL were satisfactory (see the TAAQol manual, pp. 19–21).

### Children’s coping strategies

The coping strategies of children aged 8 years or older were assessed using the Coping with a Disease Questionnaire (CODI). This questionnaire comprises 28 items, divided into six scales that measure how children and adolescents cope with their illnesses. Examples of items are “I accept my illness” (acceptance; 6), “I try to ignore my illness” (avoidance; 3), “I tell myself that even famous people have illnesses” (cognitive-palliative; 5), “I do not care about my illness” (distancing; 4), “I cry” (emotional reaction; 6), and “I hope my illness disappears” (wishful thinking; 3). Participants were asked how often they applied a particular strategy on a scale ranging from 1 (“never”) to 5 (“always”). A higher average score indicated more frequent use of a particular coping strategy. In a previous study, Cronbach’s alpha values ranged from 0.72 to 0.88 [37], while the range in values in this study was 0.47–0.94. After removing items 6, “I learn as much as possible about my illness” (cognitive-palliative) and 27, “I forget my illness” (distancing), the Cronbach’s alpha values changed from 0.47 to 0.67 and from 0.67 to 0.80, respectively [38]. These items may have lowered the internal consistency for the following reasons: Item 6 is related to gathering knowledge about the disease, while all other items assessing cognitive-palliative strategies are more focused on mind control. Item 27 refers to forgetting about the disease, while the other items are more focused on paying minimal attention to the disease.

### Parents’ coping strategies

To enable a comparison between children and parents, the CODI questionnaire was modified for parents by changing the items into questions focusing on how parents cope with their children’s illness. Examples of scale items in this adapted version were: “I accept the illness of my child” (acceptance; 6), “I try to ignore the illness of my child” (avoidance; 3), “I tell myself that even famous people have sick children” (cognitive-palliative; 5), “I do not care about the illness of my child” (distancing; 4), “I cry” (emotional reaction; 6); and “I hope the illness of my child disappears” (wishful thinking; 3). Cronbach’s alpha values ranged between 0.39 and 0.85. After the removal of items 2, “I pretend everything is alright” (avoidance), 6, “I learn as much as possible about my child’s illness” (cognitive-palliative), and 13, “I wake up at night and think of terrible things” (emotional reaction), Cronbach’s alpha values changed from 0.30 to 0.52, 0.40 to 0.59, and 0.66 to 0.72. These omitted items may have lowered the internal consistency for the following reasons: Item 2 has been removed because this item concerns general functioning, while the others are more focused on the disease. Item 6 has also been removed for the same reasons as mentioned above. At least item 13 has been deleted because this represented a more specific situation compared to the other items of the strategy emotional reaction.

### Data analysis

All of the analyses were performed using SPSS version 25. The majority of the analyses were descriptive, and the results were interpreted qualitatively because of the small sample size, which is an inherent characteristic of rare diseases such as EB. We tested the significance of associations between the quality of life and coping strategies in both children and parents, but we interpreted these results cautiously given the small sample sizes. There were missing values in the questionnaires for a number of the children and the parents, resulting in some differences in sample sizes for the different analyses. The sample sizes differed especially for the children because coping strategies were only included for children aged 8 years and above.

### Description of quality of life and coping strategies of children and parents

We first calculated the means and standard deviations for different aspects of the quality of life of children with EB and their parents. To interpret our findings, we compared the quality of life of children with EB with that of healthy children and children with other chronic diseases using data of Dutch children and toddlers derived from other studies [39, 40]. We also compared the quality of life of parents with the quality of life of healthy men and women using data presented in the TAAQOL manual (pp. 32–33). We subsequently described the coping strategies of children with EB and their parents.

### Associations between quality of life and coping strategies of children and parents

The second analytical step focused on an examination of the associations between quality of life and the use of coping strategies based on Spearman correlations. We only included parents’ emotional and social functioning because we expected these aspects to be most closely associated with coping strategies and also because we wanted to limit the number of correlations between children’s and parents’ functioning. As we were mainly interested in exploring how a certain coping strategy deployed by children increases the likelihood of their parents applying the same strategy and vice versa (e.g., greater acceptance by children associated with greater acceptance by their parents), we only calculated correlations between the same coping strategies deployed by children and parents.

## Results

The total sample consists of 55 children (28 boys and 27 girls) and 48 parents (9 men and 39 women), including 28 parent–child dyads, 27 children who participated without their parents, 20 parents who participated without their children (n = 6) or whose child was younger than 8 years (n = 14). The number of participants in each analysis differed because in some instances, only children completed the questionnaires while in others, some children and parents completed some but not all of the questionnaires. The average age of the parents was 44 years (ranging between 27 and 64 years). The parents’ educational status varied: 12% had completed high school, 30% had received intermediate vocational education, 37% had received higher vocational education, and 20% had completed university. Ethnicity of children was: Dutch (46); Mixed: Dutch and another nationality (4); European (2); Middle Eastern (2) and Asian (1). Ethnicity of participating parents was: Dutch (43); European (3); Middle Eastern (1) and Asian (1). Reasons for invitees’ nonparticipation in the study were as follows: no reason given (72%), incorrect address (10%), insufficient personal benefits from participation (10%), insufficient knowledge of the Dutch language (2%), an intellectual disability (2%), the high burden on the family (2%), and incorrect inclusion (2%).

### Quality of life and coping strategies of children and parents

The overall quality of life (Total score) of children with EB was appeared to be somewhat lower than that of the healthy group and appeared to be comparable to the quality of life of children with other chronic diseases. The only exception was the 2–4 year age group, but this group comprised only four children (see Table 1).

**Table 1:** Health-related quality of life of children with EB compared with that of children within the general population

Age group (years)	PedsQL subscale	Scores on the PedsQL Scale								
		Current study			Chronically Ill individuals			Healthy individuals		
		N	M	SD	N	M	SD	N	M	SD
2–4	Total score	4	66.85	19.67	18	79.23	22.08	275	89.16	8.51
	Physical health		64.06	24.80		80.21	25.07		92.60	9.26
	Emotional functioning		65.00	17.80		73.33	20.93		78.78	14.23
	Social functioning		67.50	21.02		81.94	21.70		90.62	12.91
	School functioning		70.83	20.97		81.94	28.04		94.84	10.35
5 –7	Total score	10	78.61	20.08	23	76.28	14.83	251	86.94	10.85
	Physical health		73.44	27.37		83.97	17.28		91.71	11.96
	Emotional functioning		71.50	23.81		68.48	18.49		78.78	16.08
	Social functioning		83.00	23.24		75.43	18.82		87.39	16.17
	School functioning		86.50	20.15		72.61	20.27		87.03	14.33
8–12	Total score	12	81.84	11.37	26	80.64	9.32	219	82.11	8.87
(self-completed)	Physical health		85.68	11.65		82.21	12.14		84.87	9.30
	Emotional functioning		80.42	14.53		78.85	13.21		77.05	13.66
	Social functioning		84.58	13.05		83.27	12.80		86.14	12.30
	School functioning		76.67	20.71		77.31	13.13		78.70	12.00
13–18	Total score	7	83.64	8.26	25	77.09	9.40	185	82.24	9.15
(self-completed)	Physical health		78.13	15.42		81.00	12.00		86.01	9.77
	Emotional functioning		91.43	8.52		71.40	16.62		76.70	15.20
	Social functioning		85.71	6.73		83.40	12.97		89.38	11.56
	School functioning		79.29	21.49		70.20	15.17		74.59	13.16
19–25	Total score	9	79.27	17.83	137	76.65	15.92	512	85.88	11.45
(self-completed)	Physical health		77.08	23.39		77.90	21.87		89.60	12.99
	Emotional functioning		77.78	18.89		72.30	18.87		78.54	17.60
	Social functioning		83.89	17.10		81.17	17.11		88.78	13.30
	School functioning		78.33	23.05		74.49	17.96		84.36	14.40

N = sample; M = mean; SD = standard deviation; Pedsql. = Pediactric Quality of Life Scale

Fathers of children with EB provided lower ratings for their quality of life for all of the sub-scales compared to fathers of children in the healthy group. Similarly, the quality of life of mothers of children with EB was lower for all of the subscales compared to mothers of healthy children, with the exception of the sex and anger scales (see Table 2).

**Table 2:** Health-related quality of life of parents of children with EB compared with healthy adults

	Current sample (N = 48)				Normative sample (N = 4386)			
	Men (N = 9)		Women (N = 39)		Men (N = 1990)		Women (N = 2396)	
	M	SD	M	SD	M	SD	M	SD
Gross motor functioning	86.1	22.0	90.5	15.2	96.9	10.3	95.5	11.3
Fine motor functioning	95.8	12.5	96.3	8.1	99.4	4.2	98.9	4.4
Cognitive functioning	89.6	16.8	78.7	27.6	90.3	16.6	88.3	19.3
Sleep	74.3	19.1	70.4	27.1	84.1	19.3	77.6	22.1
Pain	73.6	31.7	73.9	23.9	85.4	16.7	80.7	18.3
Social functioning	79.2	22.5	86.5	20.5	89.0	15.0	89.0	15.8
Daily activities	83.3	17.4	82.1	26.8	91.1	16.4	89.5	18.4
Sex	81.9	26.6	92.3	17.8	89.2	21.9	91.0	18.9
Vitality	59.3	18.4	64.1	23.0	76.0	17.7	68.8	20.4
Positive emotions	58.3	22.0	68.2	21.5	69.6	19.4	70.5	19.8
Depressive emotions	79.6	28.0	79.1	23.6	85.6	15.4	80.9	17.5
Anger	82.7	17.7	91.7	11.9	89.9	14.4	89.4	14.5

N = sample; M = mean; SD = standard deviation; Codi = Coping with a Disease Questionnaire

All children in the study reported that they generally used the same strategies more than other strategies. For example, all showed more acceptance than emotional reactions. Both children and parents deployed strategies of acceptance and wishful thinking relatively often, while emotional reactions and cognitive-palliative strategies were the least used strategies. Furthermore, children, especially older ones, appeared to use avoidance more often than their parents did (see Table 3).

**Table 3:** Coping strategies used by children with EB (by age group) and their parents

Age group (in years)	CODI subscales					
	Avoidance	Cognitive-palliative	Emotional reactions	Acceptance	Wishful thinking	Distance
	M (SD)	M (SD)	M (SD)	M (SD)	M (SD)	M (SD)
*Children*						
8–12	2.25 (1.10)	1.67 (0.48)	1.38 (0.46)	4.21 (0.92)	3.75 (1.34)	3.65 (0.70)
*N* = *12*						
13–18	3.92 (1.15)	1.68 (0.64)	1.81 (0.50)	4.48 (0.47)	4.29 (1.27)	3.47 (1.00)
*N* = *8*						
19–25	3.40 (0.89)	1.86 (0.67)	1.95 (0.72)	4.08 (0.43)	3.63 (1.41)	3.45 (1.15)
*N* = *10*						
*Parents N* = 49	1.93 (0.71)	2.53 (0.72)	1.78 (0.49)	4.21 (0.54)	3.72 (1.12)	2.69 (1.01)

N = sample; M = mean; SD = standard deviation; Codi = Coping with a Disease Questionnaire

### Associations between quality of life and coping strategies of children and parents

Children who more often used emotional reactions and cognitive-palliative strategies to cope with the disease, demonstrated lower levels of emotional and social functioning than children who used emotional reactions less frequently. Moreover, emotional, social and school functioning was reported to be better among children who showed more acceptance, while children who used distancing reported better functioning on all domains. Avoidance and wishful thinking strategies of children with EB were not related to their quality of life. Further, parents who resorted to emotional reactions demonstrated lower levels of social functioning compared to other parents while more use of avoidance was positively linked to their positive emotions. Moreover, as Table 4 indicates, emotional reactions, such as crying, were more common among parents who experienced greater degrees of depressive emotions and anger.

**Table 4:** Correlations between health-related quality of life and coping strategies

	CODI subscales					
	Avoidance	Cognitive-palliative	Emotional reactions	Acceptance	Wishful thinking	Distance
Children’s QoL (N = 28)						
Physical health	− 0.249	0.000	− 0.215	0.284	− 0.216	0.512**
Emotional functioning	− 0.151	− 0.421*	− 0.491**	0.457*	− 0.311	0.406*
Social functioning	− 0.222	− 0.070	− 0.431*	0.403*	− 0.283	0.534**
School functioning	− 0.089	− 0.090	− 0.110	0.388*	− 0.327	0.390*
Parents’ QoL (N = 48)						
Social functioning	− 0.128	− 0.026	− 0.397**	0.149	0.013	0.054
Positive emotions	0.345*	0.184	− 0.119	0.013	0.070	0.031
Depressive emotions	0.061	0.098	− 0.534**	0.031	− 0.075	0.116
Anger	− 0.004	0.081	− 0.383**	− 0.064	0.024	− 0.094

**Correlations were significant at the 0.01 level (2-tailed) and at the 0.05 level (2-tailed)

### Associations between quality of life and coping strategies within parent–child dyads

Our analysis of the relationship between the quality of life of children and parents revealed that better emotional and social functioning in children was significantly associated with less depressive emotions in the parents (N = 38; r = 0.400; p = 0.012 and r = 0.380; p = 0.017, respectively). The correlations between all other aspects of children’s quality of life and the parents’ social and emotional functioning (i.e., positive emotions, depressive emotions, and anger) ranged from − 0.190 to 0.300 (p > 0.05).

There were significant correlations between children’s frequent use of cognitive-palliative, acceptance, and distancing coping strategies and that of their parents (N = 27; r = 0.489, p = 0.010; r = 0.418, p = 0.030, and r = 0.439, p = 0.022, respectively).

Finally, parents’ frequent use of the acceptance strategy was associated with a higher level of physical functioning in their children (N = 25; r = 0.420, p = 0.037). On the other hand, children’s use of the avoidance strategy related to a lower level of anger among parents (N = 26; r = 0.410; p = 0.038).

## Discussion

Our findings indicate that the overall quality of life of children with EB and their parents was somewhat lower compared to the quality of life of healthy children and adults. Children who frequently used acceptance and distancing as coping strategies and engaged less often in emotional reactions and cognitive-palliative strategies, reported a better quality of life, while parents who frequently used emotional reactions and less often avoidance as coping strategies demonstrated a lower quality of life associated with psychosocial aspects. Children’s emotional and social functioning appeared to be negatively related to the depressive emotions of their parents, while the use of cognitive-palliative, acceptance, and distancing strategies in children and parents appeared to be mutually reinforcing. Frequent use of the acceptance strategy by parents was linked to a higher level of physical functioning in their children, while a frequent use of avoidance as a strategy by children was related to a lower level of anger among parents.

### Quality of life and coping strategies

Our finding that the overall quality of life of children with EB was somewhat lower than that of the norm group accords with those of earlier studies conducted among children with EB [13–15]. The fact that the parents’ quality of life was also lower than that of the healthy group confirms the findings of earlier studies on the burden of disease for parents of children with EB [10, 16–18].

Evidently, children and parents mostly use the same coping strategies, namely acceptance (e.g. “I am able to manage my illness”) and wishful thinking (e.g. “I hope that my illness disappears”). The chronic nature of the disease probably induces the coping strategy of acceptance because a cure is not (yet) possible, but it may also lead to wishful thinking powered by the hope that the disease will eventually heal. In addition, the findings of this study that parents deploy the avoidance strategy much less often than do children may be attributed to the necessity of providing their children with daily care, which they cannot avoid.

### Associations between quality of life and coping strategies of children and parents

It is also noteworthy that children who express emotional reactions more frequently, for example, the perception of their sickness being unfair, demonstrate lower levels of emotional and social functioning. Studies on the coping strategies of children with different disabilities also endorse the finding that emotional reactions, applied as a coping strategy, are negatively related to quality of life [37, 41]. Another notable finding is that showing emotional reactions is the only coping strategy that is negatively related to diverse domains of functioning. Further, children who are better able to accept or distance themselves of their illnesses, demonstrate better functioning to nearly all domains of functioning. A previous study has shown that children with asthma, diabetes mellitus, and arthritis who are more accepting of their illnesses report a better HRQoL [37]. In another study, acceptance and distancing were positively related to the HRQoL of children clinically diagnosed with short stature (heights that are considerably below the average height of their peers) [42]. In addition, children with chronic or acute illnesses who engaged in wishful thinking experienced higher levels of anxiety and sadness, which is not apparent from the results of this study [25].

Notably, acceptance, which was often expressed by parents, did not seem to have a positive effect on the psychosocial aspects of their quality of life. Whereas they showed relatively low levels of emotional reactions (e.g., “I cry”), more frequent use of this coping strategy was found to be associated with lower social functioning. This finding seems to accord with those of earlier studies of mothers of children with chronic diseases or physical disabilities [29, 31].

### Associations between quality of life and coping strategies within parent–child dyads

Our analysis of the relationship between children and parents revealed an association between children’s emotional and social functioning and their parents’ depressive emotions. Evidently, low emotional functioning of children, which includes feelings of fear, sadness, or anger, or low social functioning, which can include feelings of isolation or being different, are particularly worrisome for parents and affect the psychosocial aspects of their quality of life. Finally, it is notable that within parent child dyads, children’s physical functioning was linked to their parents frequent use of the acceptance strategy, while this strategy is not positively linked to their own psychosocial well-being. Children’s frequent use of the avoidance strategy was linked to a lower level of anger among parents, which to the best of our knowledge has not been shown in previous studies.

### Strengths and limitations of the study

The strength of our study is that we approached the entire population of children with EB and their parents in the Netherlands and obtained a reasonable response rate despite the burden experienced by this target group. However, the heterogeneity of EB conditions in this sample and its limited size affected the generalizability of the findings. As the small number of male parents and children in different age groups precluded us from performing statistical tests of comparison, between group differences being reported may not be significant. Moreover, its cross-sectional design impeded the discovery of causal relationships. Further, the CODI questionnaire used for the parents was a modified version that made it difficult to assess the psychometric characteristics of this questionnaire. The results of the current study can only therefore provide indications that require further research for their substantiation. Despite these limitations, this study provides new insights into the interactions of children with EB and their parents.

## Conclusions

Our findings suggest that coping strategies are related to the quality of life of children with EB and their parents. Children who are able to accept the disease or distance themselves emotionally from it appear to be better off in contrast to those who tend to show emotional reactions or cognitive-palliative strategies. The emotional and social functioning of parents who are inclined to show more emotional reactions appears to be lower than that of parents who show fewer emotional reactions, while the use of avoidance as strategy seems to be linked to positive emotions. It is important to attend to the interaction between children and their parents, as our findings indicate that [1] better emotional and social functioning in children are significantly associated with less depressive emotions in the parents, [2] children’s avoidance and parents’ acceptance coping strategies are related to the other’s quality of life, and [3] the coping strategies of children and their parents are related,. Further research is needed to investigate the causal relationships between the quality of life and coping strategies of children with EB and their parents.

## Data Availability

The data and materials can be requested from the corresponding author.

## Abbreviations

CODI: Coping with a Disease Questionnaire
DEB: Dystrophic epidermolysis bullosa
DEBRA: Dystrophic Epidermolysis Bullosa Research Association
EB: Epidermolysis bullosa
EBS: Epidermolysis bullosa simplex
HRQoL: Health-related quality of life
JEB: Junctional epidermolysis bullosa
KS: Kindler syndrome
PedsQL: Pediatric Quality of Life Inventory
RDEB: Recessive dystrophic epidermolysis bullosa
TAAQoL: TNO-AZL Questionnaire for Adult’s Health related Quality of Life
